# Effects of angiotensin receptor blockers (ARBs) on clinical outcomes of patients with hypertension and COVID-19: A 7-month follow-up cohort study

**DOI:** 10.34172/jcvtr.2022.30559

**Published:** 2022-11-26

**Authors:** Azar Hadadi, Sina Kazemian, Mahan Shafie, Arezoo Ahmadi, Abbas Soleimani, Haleh Ashraf

**Affiliations:** ^1^Research Center for Clinical Virology, Tehran University of Medical Sciences, Tehran, Iran; ^2^Cardiac Primary Prevention Research Center, Cardiovascular Diseases Research Institute, Tehran University of Medical Sciences, Tehran, Iran; ^3^School of Medicine, Tehran University of Medical Sciences, Tehran, Iran; ^4^NeuroTRACT Association, Students’ Scientific Research Center, Tehran University of Medical Sciences, Tehran, Iran; ^5^Department of Anesthesiology and Critical Care, Sina Hospital, Faculty of Medicine, Tehran University of Medical Sciences, Tehran, Iran; ^6^Department of Cardiology, Sina Hospital, Tehran University of Medical Sciences, Tehran, Iran; ^7^Rajaie Cardiovascular Medical and Research Center, Iran University of Medical Sciences, Tehran, Iran

**Keywords:** Angiotensin-Converting Enzyme Inhibitors, COVID-19, Hypertension, Renin-Angiotensin System, SARS-CoV-2

## Abstract

***Introduction:*** Since the coronavirus disease 2019 (COVID-19) pandemic, the use of angiotensin II receptor blockers (ARBs) in hypertensive patients with COVID-19 has been controversial. Following our previous study, after one year, we intended to extend our sample size and results to investigate the effects of ARBs with both in-hospital outcomes and 7-month follow-up results in patients with COVID-19.

***Methods:*** Patients with a diagnosis of COVID-19 who were admitted to Sina Hospital, Tehran, Iran, from February to October 2020 participated in this follow-up cohort study. The COVID-19 diagnosis was based on a positive polymerase chain reaction test or chest computed tomography scan according to guidelines. Patients were followed for disease severity, incurring in-hospital mortality, complications, and 7-month all-cause mortality.

***Results:*** We evaluated 1413 patients with COVID-19 in this study. After excluding 124 patients, 1289 including 561(43.5%) hypertensive patients, entered the analysis. During the study, 875(67.9%) severe disease, 227(17.6%) in-hospital mortality, and 307(23.8%) 7-month all-cause mortality were observed. After adjusting for possible confounders, ARB was not associated with severity, in-hospital and 7-month all-cause mortality, and in-hospital complications except for acute kidney injury. Discontinuation of ARBs was significantly associated with higher in-hospital mortality and 7-month all-cause mortality (both *P* values<0.006). We observed a better 7-month outcome in those who continued their ARBs after discharge.

***Conclusion:*** The results of this study, along with the previous studies, provide reassurance that taking ARBs is not associated with the risk of mortality, complications, and poorer outcomes in hypertensive COVID-19 patients after adjustment for possible confounders.

## Introduction

 Since coronavirus disease-2019 (COVID-19), caused by the severe acute respiratory syndrome coronavirus 2 (SARS-CoV-2) was first discovered in Wuhan, China, in December 2019, many studies have suggested cardiovascular risk factors such as hypertension and diabetes as significant risk factors for severity or susceptibility to COVID-19.^1,2^ It has been suggested that angiotensin-converting enzyme 2 (ACE2) has a predominant role in the pathogenesis of COVID-19, considering that ACE2 could be the target receptor of SARS-CoV-2 and facilitate virus entry and replication in the host cell.^3^ Therefore, it appears that the renin-angiotensin-aldosterone system (RAAS) could be associated with the disease process, and there have been concerns about the risk of RAAS inhibitors usage in COVID-19 patients, which may lead to upregulation, increased expression, and presentation of ACE2 in the different tissues.^4^ Accordingly, the use of angiotensin-converting enzyme inhibitors (ACEIs) and angiotensin II receptor blockers (ARBs) in patients with COVID-19 and hypertension was a great concern, especially very early in the pandemic.^5^ Although several studies have evaluated the association between these medications and in-hospital, there are still clinical uncertainties about maintaining or discontinuing these medications in hypertensive patients diagnosed with COVID-19, and there is no report on long-term outcomes on this topic.^6-8^

 Our previous study investigated the association of ARBs usage with in-hospital outcomes in patients with COVID-19. We concluded that taking ARBs in hypertensive patients diagnosed with COVID-19 is not associated with poorer in-hospital outcomes.^9^ After one year, we intended to extend our data and sample size to investigate the association of ARBs with both in-hospital outcomes and 7-month follow-up mortality in patients with COVID-19.

## Materials and Methods

 We included patients with age ≥ 18 years and COVID-19 diagnosis who were admitted to Sina Hospital affiliated with the Tehran University of Medical Sciences from February to October 2020. The algorithm of clinical care for patients presenting with respiratory symptoms to the Sina hospital emergency department has previously been reported.^10^ The COVID-19 diagnosis was based on a positive polymerase chain reaction test for SARS-CoV-2 from oropharyngeal or throat swab or consolidation in chest computed tomography (CT) scan, which could not be explained by other etiologies and confirmation of two radiologists independently, based on the WHO’s interim guidance^11^ and the guideline of Iranian National Committee of COVID-19.^12^

 We defined severe COVID-19 according to CDC criteria, and all in-hospital complications were determined based on previously published reports.^9,13,14^ Hypertension was defined as systolic blood pressure ≥ 140 mm Hg, diastolic blood pressure ≥ 90 mm Hg, or antihypertensive treatment. All demographics and clinical data were gathered from hospital medical records by trained medical staff. The 7-month all-cause mortality data was obtained through monthly telephone calls after discharge and through death time records in National Organization for Civil Registration linked with hospital data for the whole cohort. The participants were followed for a mean duration of 215.2 days (range: 129-366 days).

 We presented categorical variables as numbers (%) and compared them using the chi-square and Fisher exact test. The numerical variables’ normality was evaluated using the Shapiro-Wilk and Kolmogorov–Smirnov tests. Normally distributed variables were reported as mean ± standard deviation and compared using the independent group t-test. Besides, variables with skewed distribution were presented as median [interquartile range] and compared using the Mann-Whitney U-test. In addition, we categorized patients with hypertension into 4 groups based on the history of ARB usage during hospitalization: A) Continued group: patients who continued taking ARBs at least for 7 days after admission. B) Discontinued group: patients who discontinued using ARBs within 7 days after admission. C) Newly started group: patients who were newly started on taking an ARB after hospitalization. D) Never used group: patients who never used any ARB. The most common reason for discontinuation of ARB was the inclusion in the trial in 38 (69.1%),^15^ both AKI and shock in 8 (14.5%), AKI in 6 (10.9%), and shock in 3 (5.5%) patients. We fitted multivariable binary regression models to evaluate the prognostic value of ARB for predicting clinical outcomes. Variables with clinical significance or *P* < 0.1 in univariate regression were considered possible confounders (including age, sex, diabetes mellitus, cardiac disease, cerebrovascular disease, chronic lung disease, chronic kidney disease, and malignancy) and entered the multivariable regression models. All statistical analyses were performed using the SPSS 21.0 software, and *P* value ≤ 0.05 was considered statistically significant.

## Results

 We evaluated 1413 patients with a diagnosis of COVID-19 from February 2020 to October 2020. After excluding 124 patients (90 patients due to lack of key information and 34 due to a history of ACE inhibitors usage), 1289 patients, including 561 hypertensive patients, entered the final analysis. The mean age was 60.00 ± 16.54 years, and 789(61.2%) were male. The most common comorbidities were hypertension (43.5%), diabetes (29.8%), and cardiac disease (22.7%). During the study, 875(67.9%) patients manifested severe disease, and 227(17.6%) died during the in-hospital course. The 7-month all-cause mortality was 307(23.8%) patients.

 A total number of 241(42.96%) hypertensive patients had a history of taking ARBs (Losartan: N = 209 and Valsartan: N = 32). Patients with a history of ARB usage were more likely to be older, have diabetes, have cardiac disease, receive cardiovascular medications, incur severe COVID-19, and develop AKI compared to non-ARB users (Supplementary File, Table S1). Demographic and baseline characteristics of disease severity and 7-month all-cause mortality are presented in (Supplementary File, Table S2). During the unadjusted evaluation, older age, history of chronic lung disease, higher admission neutrophil to lymphocyte ratio, urea, C reactive protein, lactate dehydrogenase, and aspartate transaminase were associated with both severe and 7-month all-cause mortality. In addition, female sex and history of diabetes increased the severity risk, and positive history of cerebrovascular disease and malignancy was only associated with an increased risk of 7-month mortality.

 We used multivariable regression models in both all patients and hypertensive patients populations to evaluate the prognostic value of ARB usage for predicting clinical outcomes (Table 1). After adjustment with possible confounders, ARB usage was independently associated with AKI development in both hypertensive patients (odds ratio (OR): 1.65, 95% confidence interval (CI): 1.05-2.59, *P* value: 0.031) and whole cohort population (OR: 1.63, 95% CI: 1.04-2.55, *P* value: 0.034) models. We have found that taking ARBs is not associated with severity, in-hospital or 7-month mortality, or in-hospital complications except for AKI in both models.

**Table 1:** Prognostic value of ARB usage for prediction of clinical outcomes in patients with COVID-19

**Clinical outcomes**	**All patients** ^a^			**Hypertensive patients** ^b^		
	**OR**	**95% CI**	***P***	**OR**	**95% CI**	***P***
Severity	1.45	0.97-2.18	0.073	1.45	0.96-2.19	0.078
In-hospital mortality	1.12	0.75-1.69	0.574	1.14	0.75-1.73	0.534
7-month all-cause mortality	0.74	0.50-1.10	0.131	0.75	0.50-1.22	0.162
ARDS	1.29	0.90-1.86	0.165	1.29	0.89-1.87	0.175
Invasive ventilation	1.19	0.76-1.88	0.451	1.21	0.76-1.92	0.426
ACI	1.06	0.72-1.56	0.765	1.10	0.75-1.62	0.627
AKI	1.63	1.04-2.55	0.034*	1.65	1.05-2.59	0.031*
ALI	0.91	0.53-1.55	0.731	0.89	0.52-1.53	0.679
Multiorgan damage	1.18	0.80-1.74	0.401	1.20	0.80-1.78	0.373

Abbreviations: ACI, acute cardiac injury; AKI: acute kidney injury; ALI: acute liver injury; ARDS: acute respiratory distress syndrome; OR, odds ratio; CI, confidence interval. ^a^ Multivariate logistic regression adjusted for age, sex, diabetes mellitus, hypertension, cardiac disease, cerebrovascular disease, chronic lung disease, chronic kidney disease, and malignancy. ^b^ Multivariate logistic regression adjusted for age, sex, diabetes mellitus, cardiac disease, cerebrovascular disease, chronic lung disease, chronic kidney disease, and malignancy. * Statistically significant.

 In subgroup analysis based on the history of ARBs usage, patients who never used ARBs were more likely to be younger, male, and with a negative history of diabetes mellitus and cardiac disease (Supplementary File,Table S3). Moreover, discontinuation of ARBs was significantly associated with higher in-hospital mortality 24(45.5%) and 7-month all-cause mortality 28(20.9%) compared to other groups (both *P* values < 0.006). The continuation of ARBs after discharge was significantly linked with lower 7-month all-cause mortality 43(23.1%), and patients who never used ARBs were at lower risk for developing AKI during their in-hospital course 28(12.3%) (Supplementary File,Table S4).

## Discussion

 In this study, we have found that the history of ARB usage in patients with hypertension and COVID-19 was not associated with 7-month all-cause mortality, in-hospital mortality, severity, or in-hospital complications except for AKI compared to non-ARB users. We have found a significantly higher in-hospital and 7-month all-cause mortality in patients who discontinued their ARBs during hospitalization. Moreover, we observed a better 7-month outcome in those who continued their ARBs after discharge.

 The discovery of SARS-CoV-2 has put ACE2 in the spotlight. Besides its physiological functions, such as negative regulator of the RAAS and transporter action for amino acids, it is the main cell entry receptor for SARS-CoV-2. This enzyme negatively regulates tissue angiotensin II levels by breaking it into angiotensin 1-7, which has protective effects with anti-inflammation and anti-fibrosis effects after binding to Mas receptors. Therefore, ACE2 can potentially attenuate the acute inflammation response in different organs by its antagonizing effect on angiotensin II. On the other hand, there is some evidence that therapeutic doses of RAAS blockers in humans may lead to upregulation and overexpression of ACE2 at the cell surface, increasing the susceptibility to COVID-19.^4^ Recently, Hamet et al also suggested an association between ACE2 polymorphism and severity of outcomes to COVID-19. The T allele of the single nucleotide polymorphism rs2074192 of the ACE2 gene was a predisposing risk factor for earlier hypertension in obese, smoking males. They experienced more severe COVID-19 compared to other groups.^16^ Despite recent studies, there is controversy about the effects of renin-angiotensin inhibitors with COVID-19, which caused uncertainties in clinical practice.

 In our previous report, we concluded that taking ARBs is not independently associated with poorer in-hospital outcomes, except for AKI.^9^ In this study, with a larger sample size and 7-month follow-up results, we observed that taking ARBs did not increase the risk of 7-month all-cause mortality, in-hospital mortality, and complications except for AKI, which is in line with previous findings. In a study by Dublin et al there was no association between using different doses of RAAS inhibitors and the risk of COVID-19 or admission rate.^17^

 We observed that taking ARB was independently associated with a higher incidence of AKI in both hypertensives (OR: 1.65, 95% CI: 1.05-2.59) and all patients (OR: 1.63, 95% CI: 1.04-2.55) models. This finding may be attributed to the adverse events of ARBs, as reported before.^18^ This finding indicates a necessity for close observation and a conservative approach for fluid therapy in COVID-19 patients with a history of ARB usage to avoid AKI.

 Continuing or discontinuing ARBs in hypertensive patients with COVID-19 was one of the topics of interest since the beginning of the COVID-19 pandemic. After subgroup analysis without adjustment, we found that discontinuing ARBs after hospitalization was associated with significantly higher in-hospital and 7-month all-cause mortality. At the same time, patients who continued their medication had significantly lower 7-month all-cause mortality compared to others. The incidence of AKI was also significantly lower in patients who never used ARBs. AKI is a known complication after the long-term use of ARBs; however, the most common reason for ARBs discontinuation in our study was the inclusion in a trial study.^15^ Besides, we observed a similar baseline creatinine level and AKI rate among patients who continued or discontinued taking ARBs after admission. These findings show that a slightly higher rate of AKI in patients who discontinued using ARBs cannot explain significantly higher 7-months follow-up mortality alone. These findings contradict three recent clinical trial studies on this subject.^15,19,20^ These trials demonstrated a similar intensive care unit admission rate, admission duration, in-hospital mortality, and major complications between discontinued and continued ACEIs/ARBs medications.^15,19,20^ We believe that non-random analysis, confounding factors such as age or gender, different study populations, and sample sizes may be responsible for these different outcomes in patients who discontinue their medications after hospitalization.

 Notwithstanding some strengths, including its long-term follow-up and focus on ARBs rather than combined ACEIs/ARBs, important limitations must be considered. First, it is an observational study with potential biases. Second, further multicentral studies with different ethnicities are warranted to confirm the results. Third, we did not know the continuation or discontinuation of these medications after discharge, and since we did not know the exact cause of death, we used 7-month all-cause mortality.

## Conclusion

 In conclusion, the results of this study, along with all studies reported so far, provide reassurance that using ARBs is not associated with the risk of 7-month mortality and poorer outcome in hypertensive COVID-19 patients. Our results support the recommendations of cardiology societies to continue treatment with their antihypertensive medications.^21^

## Acknowledgments

 We acknowledge all healthcare workers involved in the diagnosis and treatment of patients in Sina Hospital. We are indebted to the Research Development Center of Sina Hospital for its support.

## Author Contributions

**Conceptualization: **Azar Hadadi, Sina Kazemian, Haleh Ashraf.

**Methodology: **Sina Kazemian, Haleh Ashraf.

**Formal Analysis:** Sina Kazemian, Mahan Shafie.

**Investigation: **Azar Hadadi, Arezoo Ahmadi, Abbas Soleimani.

**Resources: **Azar Hadadi, Arezoo Ahmadi, Abbas Soleimani.

**Data Curation: **Sina Kazemian, Haleh Ashraf.

**Writing—Original Draft Preparation: **Sina Kazemian, Mahan Shafie.

**Writing—Review and Editing: **Azar Hadadi, Arezoo Ahmadi, Abbas Soleimani.

**Supervision: **Haleh Ashraf.

## Funding

 Tehran University of Medical Sciences supported this study. The funding source had no role in the study design, data collection, data analysis, data interpretation, writing of the manuscript, or submission decision.

## Ethical Approval

 This study was approved by the Ethics Committee at Tehran University of Medical Science (IR.TUMS.VCR.REC.1399.018). All participants gave written informed consent before inclusion in the study.

## Competing Interests

 The authors have no conflicts of interest to declare.

## Supplementry Files

Supplementary file contains Table S1, Table S2,Table S3, Table S4.
