# Supplementary material for: Effects of angiotensin receptor blockers (ARBs) on clinical outcomes of patients with hypertension and COVID-19: A 7-month follow-up cohort study
Source: J Cardiovasc Thorac Res. 2022 Nov 26;14(4):253–7. doi: 10.34172/jcvtr.2022.30559 (PMC9871165; doi:10.34172/jcvtr.2022.30559)
Supplement: Supplementary file 1 — Supplementary file contains Table S1, Table S2,Table S3, Table S4. [file jcvtr-14-253-s001.pdf]

J Cardiovasc Thorac Res, 2022, 14(4), 253-257
doi: 10.34172/jcvtr.2022.30559
http://jcvtr.tbzmed.ac.ir

Effects of angiotensin receptor blockers (ARBs) on clinical outcomes of patients with hypertension and COVID-19: A 7-month follow-up cohort study

Azar Hadadi[1#], Sina Kazemian[2#], Mahan Shafie[3,4], Arezoo Ahmadi[5], Abbas Soleimani[6], Haleh Ashraf[2,7*]

[1]Research Center for Clinical Virology, Tehran University of Medical Sciences, Tehran, Iran
[2]Cardiac Primary Prevention Research Center, Cardiovascular Diseases Research Institute, Tehran University of Medical Sciences, Tehran, Iran
[3]School of Medicine, Tehran University of Medical Sciences, Tehran, Iran
[4]NeuroTRACT Association, Students' Scientific Research Center, Tehran University of Medical Sciences, Tehran, Iran
[5]Department of Anesthesiology and Critical Care, Sina Hospital, Faculty of Medicine, Tehran University of Medical Sciences, Tehran, Iran
[6]Department of Cardiology, Sina Hospital, Tehran University of Medical Sciences, Tehran, Iran
[7]Rajaie Cardiovascular Medical and Research Center, Iran University of Medical Sciences, Tehran, Iran

Supplementary

Table S1. Baseline characteristics and clinical outcomes of hypertensive patients with and without ARB treatment

Characteristic*		Total (N=561)	ARB users (N=241)	Non-ARB users (N=320)	P†
**Demographics**					
Age (year)		66.4±13.7	68.3±12.3	65.0±14.5	**0.014**
Sex	Female	235 (41.9%)	112 (46.5%)	123 (38.4%)	0.056
	Male	326 (58.1%)	129 (53.5%)	197 (61.6%)	
**Comorbidities**					
DM		248 (44.2%)	119 (49.4%)	129 (40.3%)	**0.032**

Cardiac disease	227 (40.5%)	121 (50.2%)	106 (33.1%)	**<0.001**
Cerebrovascular disease	48 (8.6%)	22 (9.1%)	26 (8.1%)	0.674
Chronic lung disease	37 (6.6%)	14 (5.8%)	23 (7.2%)	0.515
Chronic kidney disease	48 (8.6%)	27 (11.2%)	21 (6.6%)	0.052
Malignancy	21 (3.7%)	8 (3.3%)	13 (4.1%)	0.646
**Drug history**				
Statin	135 (24.1%)	105 (43.6%)	30 (9.4%)	**<0.001**
Aspirin	150 (26.7%)	112 (46.5%)	38 (11.9%)	**<0.001**
Metformin	113 (20.1%)	66 (27.4%)	47 (14.7%)	**<0.001**
Beta blocker	129 (23.0%)	85 (35.3%)	44 (13.8%)	**<0.001**
Calcium channel blocker	68 (12.1%)	49 (20.3%)	19 (5.9%)	**<0.001**
**Baseline laboratory data**				
WBC ($x10^9$/L)	7.2 [5.4-9.8]	6.7 [5.1-9.7]	7.6 [5.5-9.9]	0.080
Neutrophil ($x10^9$/L)	5.5 [3.7-8.1]	5.3 [3.6-7.8]	5.9 [3.8-8.3]	0.089
Lymphocyte ($x10^9$/L)	1.1 [0.8-1.6]	1.1 [0.7-1.7]	1.1 [0.2-1.6]	0.460
Platelets ($x10^9$/L)	191.0 [149.0-263.0]	189.0 [148.0-267.0]	191.0 [149.0-262.0]	0.979
Neutrophil-to-lymphocyte ratio	4.7 [2.8-8.8]	4.7 [2.7-8.6]	4.8 [3.0-9.2]	0.562
Platelet-to-lymphocyte ratio	173.8 [119.3-259.3]	181.6 [123.3-276.3]	165.3 [116.0-256.5]	0.316
SII	913.6 [495.5-1814.9]	909.3 [500.4-1804.8]	914.3 [490.7-1843.4]	0.817

RBC (x10^{12}/L)	4.6 [4.1-5.0]	4.5 [3.9-5.0]	4.6 [4.1-5.1]	**0.007**
Hemoglobin (g/dL)	13.2 [11.0-14.8]	13.0 [11.4-14.7]	13.3 [12.1-14.9]	**0.022**
Urea (mg/dL)	41.5 [29.0-66.0]	46.0 [30.0-74.0]	39.0 [28.0-63.0]	**0.038**
Creatinine (mg/dL)	1.2 [1.0-1.5]	1.2 [1.0-1.7]	1.1 [0.9-1.4]	**0.018**
BUN/Creatinine	16.2 [12.4-21.0]	16.4 [12.5-20.5]	16.0 [12.3-21.3]	0.801
Sodium (mmol/L)	136.3 [132.5-139.6]	136.0 [132.1-139.5]	136.4 [132.9-139.8]	0.428
Potassium (mmol/L)	4.4 [4.0-4.7]	4.4 [4.0-4.7]	4.3 [4.0-4.7]	0.559
CRP (mg/L)	64.9 [29.3-101.8]	65.4 [30.8-103.5]	64.3 [27.5-99.0]	0.281
ESR (mm/h)	53.0 [32.0-85.0]	56.0 [35.0-86.0]	50.0 [28.0-84.0]	0.102
LDH (U/L)	606.0 [480.0-779.0]	600.5 [472.2-749.0]	613.0 [492.0-805.0]	0.491
hs-cTnI (pg/mL)	7.5 [2.4-28.2]	7.9 [3.0-33.8]	7.0 [1.9-24.3]	0.223
AST (U/L)	54.0 [39.0-73.0]	55.0 [38.0-72.5]	54.0 [40.0-74.0]	0.585
ALT (U/L)	38.0 [28.0-54.0]	36.0 [27.0-48.7]	39.0 [29.0-59.0]	**0.011**
ALP (U/L)	176.0 [140.0-236.0]	176.0 [138.0-229.0]	177.0 [142.0-237.0]	0.484
**Outcomes**				
Hospital length of stay (day)	5.0 [3.0-9.0]	6.0 [3.0-9.0]	5.0 [3.0-9.0]	**0.016**
Severity	421 (75.0%)	192 (79.7%)	229 (71.6%)	**0.028**
In-hospital mortality	135 (24.1%)	63 (26.1%)	72 (22.5%)	0.318
7-month all-cause mortality	176 (31.4%)	71 (29.5%)	105 (32.8%)	0.397
ARDS	185 (33.0%)	89 (36.9%)	96 (30.0%)	0.084

Invasive ventilation	97 (17.3%)	46 (19.1%)	51 (15.9%)	0.329
ACI	167 (29.8%)	78 (32.4%)	89 (27.8%)	0.243
AKI	102 (18.2%)	57 (23.7%)	45 (14.1%)	**0.004**
ALI	65 (11.6%)	28 (11.6%)	37 (11.6%)	0.984
Multiorgan damage	150 (26.7%)	72 (29.9%)	78 (24.4%)	0.145

Abbreviations: ACI: acute cardiac injury; AKI: acute kidney injury; ALI: acute liver injury; ALP: alkaline phosphatase; ALT: alanine aminotransferase; ARB: angiotensin II receptor blockers; ARDS: acute respiratory distress syndrome; AST: aspartate aminotransferase; BUN: blood urea nitrogen; CRP: C-reactive protein; DM: diabetes mellitus; ESR: erythrocyte sedimentation rate; hs-cTnI: high-sensitivity cardiac troponin I; LDH: lactate dehydrogenase; RBC: red blood cells; SII: systemic immune-inflammation index; WBC: white blood cells.

* Data are presented as mean±standard deviation, number (%), or median [interquartile range].

† Statistically significant P-values are bolded.

Table S2. Disease severity and 7-month all-cause mortality rates of COVID-19 in hypertensive patients.

Characteristic*		Severity			7-month all-cause mortality		
		**Severe (N=421)**	**Non-severe (N=140)**	**P†**	**Deceased (N=176)**	**Survived (N=385)**	**P†**
**Demographics**							
Age (years)		67.6±13.2	62.9±14.3	**0.001**	72.9±11.6	63.5±13.5	**<0.001**
Sex	Female	189 (44.9%)	46 (32.9%)	**0.012**	76 (43.2%)	159 (41.3%)	0.675
	Male	232 (55.1%)	94 (67.1%)		100 (56.8%)	226 (58.7%)	
**Comorbidities**							
DM		199 (47.3%)	49 (35.0%)	**0.011**	79 (44.9%)	169 (43.9%)	0.827
Cardiac disease		173 (41.1%)	54 (38.6%)	0.599	78 (44.3%)	149 (38.7%)	0.208
Cerebrovascular disease		40 (9.5%)	8 (5.7%)	0.165	27 (15.3%)	21 (5.5%)	**<0.001**
Chronic lung disease		33 (7.8%)	4 (2.9%)	**0.040**	17 (9.7%)	20 (5.2%)	**0.048**
Chronic kidney disease		37 (8.8%)	11 (7.9%)	0.733	20 (11.4%)	28 (7.3%)	0.108
Malignancy		17 (4.0%)	4 (2.9%)	0.524	12 (6.8%)	9 (2.3%)	**0.009**
**Drug history**							
Statin		112 (26.6%)	23 (16.4%)	**0.015**	37 (21.0%)	98 (25.5%)	0.255
Aspirin		116 (27.6%)	34 (24.3%)	0.449	45 (25.6%)	105 (27.3%)	0.672
Metformin		92 (21.9%)	21 (15.0%)	0.080	34 (19.3%)	79 (20.5%)	0.742

Beta blocker	104 (24.7%)	25 (17.9%)	0.095	46 (26.1%)	83 (21.6%)	0.232
ARB	192 (45.6%)	49 (35.0%)	**0.028**	71 (40.3%)	170 (44.2%)	0.397
Calcium channel blocker	55 (13.1%)	13 (9.3%)	0.235	22 (12.5%)	46 (11.9%)	0.853
**Baseline laboratory data**						
WBC (x10^9/L)	7.5 [5.4-9.9]	7.0 [5.2-9.3]	0.211	8.8 [5.8-12.4]	6.7 [5.2-9.2]	**<0.001**
Neutrophil (x10^9/L)	5.7 [3.9-8.2]	4.8 [3.3-7.4]	**0.016**	6.9 [4.4-9.7]	5.0 [3.6-7.4]	**<0.001**
Lymphocyte (x10^9/L)	1.1 [0.7-1.5]	1.3 [0.9-1.8]	**<0.001**	1.0 [0.7-1.4]	1.2 [0.9-1.7]	**0.003**
Platelets (x10^9/L)	191.0 [148.0-273.0]	187.0 [150.0-255.0]	0.667	189.0 [144.0-264.5]	192.0 [150.0-263.0]	0.590
Neutrophil-to-lymphocyte ratio	5.2 [3.1-9.5]	3.6 [2.4-6.2]	**<0.001**	6.5 [4.1-10.8]	4.0 [2.6-7.3]	**<0.001**
Platelet-to-lymphocyte ratio	188.3 [124.4-276.6]	140.4 [110.6-201.0]	**<0.001**	196.7 [124.6-285.0]	165.4 [118.3-251.6]	**0.049**
SII	988.7 [521.6-1931.4]	724.0 [459.9-1290.8]	**<0.001**	1294.2 [642.4-2350.7]	817.5 [470.2-1570.2]	**<0.001**
RBC (x10^{12}/L)	4.5 [4.0-5.0]	4.7 [4.1-5.1]	0.087	4.4 [3.9-4.9]	4.6 [4.1-5.1]	**<0.001**
Hemoglobin (g/dL)	13.1 [11.8-14.6]	13.8 [12.3-15.0]	0.058	12.8 [10.7-14.4]	13.4 [12.1-14.9]	**<0.001**
Urea (mg/dL)	45.0 [30.0-71.0]	36.0 [27.0-54.0]	**0.009**	59.0 [36.0-98.0]	37.0 [26.0-54.0]	**<0.001**
Creatinine (mg/dL)	1.2 [1.0-1.6]	1.4 [1.1-1.4]	0.137	1.3 [1.0-1.9]	1.1 [0.9-1.4]	**<0.001**

BUN/Creatinine	16.9 [12.6-21.5]	15.0 [11.6-18.4]	**0.007**	18.9 [14.4-25.0]	15.3 [11.8-19.5]	**<0.001**
Sodium (mmol/L)	135.9 [132.0-139.0]	137.2 [134.3-141.0]	**0.001**	135.9 [132.3-139.7]	136.4 [132.5-139.5]	0.635
Potassium (mmol/L)	4.4 [4.0-4.7]	4.3 [4.0-4.6]	0.155	4.5 [3.9-4.9]	4.3 [4.0-4.7]	0.303
CRP (mg/L)	73.2 [38.6-103.8]	41.4 [10.5-75.4]	**<0.001**	95.0 [49.0-135.3]	57.7 [24.4-92.0]	**<0.001**
ESR (mm/h)	56.0 [35.0-87.5]	42.0 [25.0-76.5]	**<0.001**	59.0 [35.0-85.5]	50.0 [30.0-85.0]	0.113
LDH (U/L)	633.0 [516.0-800.0]	499.5 [390.7-632.2]	**<0.001**	691.0 [531.0-896.7]	577.0 [467.0-741.0]	**<0.001**
hs-cTnI (pg/mL)	8.7 [3.0-37.0]	4.3 [1.6-11.0]	**<0.001**	25.6 [5.8-86.4]	5.7 [1.7-13.9]	**<0.001**
AST (U/L)	57.0 [43.0-76.0]	40.0 [33.0-55.7]	**<0.001**	62.5 [42.0-80.0]	49.0 [38.0-67.0]	**<0.001**
ALT (U/L)	40.0 [29.0-56.7]	34.5 [25.0-45.0]	**0.001**	41.5 [29.0-58.0]	37.0 [28.0-52.0]	0.069
ALP (U/L)	177.0 [140.0-237.0]	175.0 [140.5-220.0]	0.936	192.0 [148.0-270.0]	172.0 [138.0-222.0]	**0.002**
**Outcomes**						
Hospital length of stay (day)	6.0 [3.0-10.]	4.0 [2.0-7.0]	**<0.001**	7.0 [3.0-11.0]	5.0 [3.0-8.0]	**0.002**
ARDS	181 (43.0%)	4 (2.9%)	**<0.001**	114 (64.8%)	71 (18.4%)	**<0.001**
Invasive ventilation	93 (22.1%)	4 (2.9%)	**<0.001**	93 (52.8%)	4 (1.0%)	**<0.001**

ACI	147 (34.9%)	20 (14.3%)	**<0.001**	92 (52.3%)	75 (19.5%)	**<0.001**
AKI	93 (22.1%)	9 (6.4%)	**<0.001**	72 (40.9%)	30 (7.8%)	**<0.001**
ALI	53 (12.6%)	12 (8.6%)	0.198	34 (19.3%)	31 (8.1%)	**<0.001**
Multiorgan damage	141 (33.5%)	9 (6.4%)	**<0.001**	105 (59.7%)	45 (11.7%)	**<0.001**

Abbreviations: ACI: acute cardiac injury; AKI: acute kidney injury; ALI: acute liver injury; ALP: alkaline phosphatase; ALT: alanine aminotransferase; ARB: angiotensin II receptor blockers; ARDS: acute respiratory distress syndrome; AST: aspartate aminotransferase; BUN: blood urea nitrogen; CRP: C-reactive protein; DM: diabetes mellitus; ESR: erythrocyte sedimentation rate; hs-cTnI: high-sensitivity cardiac troponin I; LDH: lactate dehydrogenase; RBC: red blood cells; SII: systemic immune-inflammation index; WBC: white blood cells.

* Data are presented as mean±standard deviation, number (%), or median [interquartile range].

† Statistically significant P-values are bolded.

Table S3. Baseline characteristics of hypertensive patients with COVID-19 based on the history of ARB usage.

Characteristic*		Continued (N=186)	Discontinued (N=55)	Newly started (N=93)	Never used (N=227)	P†
**Demographics**						
Age (year)		67.3±12.4	71.1±11.6	67.8±12.0	63.8±15.2‡	**0.001**
Sex	Female	88 (47.3%)	24 (43.6%)	44 (47.3%)	79 (34.8%)‡	**0.044**
	Male	98 (52.7%)	31 (56.4%)	49 (52.7%)	148 (65.2%)	
**Comorbidities**						
DM		92 (49.5%)	27 (49.1%)	52 (55.9%)	77 (33.9%)‡	**0.001**
Cardiac disease		91 (48.9%)	30 (54.5%)	45 (48.4%)	61 (26.9%)‡	**<0.001**
Cerebrovascular disease		17 (9.1%)	5 (9.1%)	6 (6.5%)	20 (8.8%)	0.886
Chronic lung disease		10 (5.4%)	4 (7.3%)	7 (7.5%)	16 (7.0%)	0.874
Chronic kidney disease		22 (11.8%)	5 (9.1%)	8 (8.6%)	13 (5.7%)	0.180
Malignancy		6 (3.2%)	2 (3.6%)	1 (1.1%)	12 (5.3%)	0.324
**Baseline laboratory data**						
WBC (x10^9/L)		6.5 [5.1-8.8]	9.3 [6.0-12.1]	7.0 [5.5-9.6]	7.9 [5.5-10.0]	**<0.001**
Neutrophil (x10^9/L)		4.7 [3.4-7.3]	7.4 [4.7-10.2]	5.5 [3.6-7.8]	6.0 [3.9-8.8]	**<0.001**
Lymphocyte (x10^9/L)		1.0 [0.8-1.5]	1.3 [0.7-2.0]	1.1 [0.8-1.5]	1.2 [0.8-1.6]	0.459
Platelets (x10^9/L)		187.5 [143.7-268.2]	215.0 [173.0-266.0]	190.0 [150.7-271.0]	192.0 [148.0-260.5]	0.732
Neutrophil-to-		4.6 [2.5-8.2]	5.1 [3.4-10.3]	4.5 [2.6-7.0]	5.0 [3.1-9.4]	0.172

lymphocyte ratio					
Platelet-to-lymphocyte ratio	186.6 [127.3-279.0]	142.6 [114.9-275.5]	175.1 [118.2-249.5]	159.9 [115.6-259.1]	0.419
SII	909.3 [490.1-1734.8]	989.9 [581.5-2323.4]	464.5 [810.1-1729.9]	933.7 [502.0-1914.3]	0.414
RBC (x10^{12}/L)	4.4 [4.0-4.9]	4.6 [3.9-5.0]	4.5 [4.0-5.0]	4.7 [4.2-5.2]	**0.007**
Hemoglobin (g/dL)	13.0 [11.4-14.6]	13.1 [11.2-14.8]	12.9 [11.6-14.4]	13.6 [12.3-15.0]	**0.008**
Urea (mg/dL)	44.0 [30.0-66.2]	53.0 [32.0-89.0]	44.0 [30.7-66.5]	38.0 [26.0-59.0]	**0.017**
Creatinine (mg/dL)	1.2 [1.0-1.6]	1.2 [1.0-1.8]	1.1 [0.9-1.5]	1.1 [0.9-1.4]	0.117
BUN/Creatinine	16.0 [12.3-19.8]	19.1 [13.3-22.8]	17.2 [12.9-22.4]	15.6 [11.5-21.2]	**0.046**
Sodium (mmol/L)	135.4 [131.7-139.3]	136.9 [134.0-139.8]	136.4 [132.2-139.0]	136.4 [133.3-140.2]	0.195
Potassium (mmol/L)	4.4 [4.0-4.7]	4.5 [4.0-4.8]	4.4 [4.1-4.9]	4.3 [3.9-4.7]	0.235
CRP (mg/L)	65.4 [27.7-102.6]	68.4 [42.1-126.4]	70.4 [40.0-103.6]	59.8 [23.9-96.7]	0.262
ESR (mm/h)	56.0 [36.0-88.0]	53.0 [34.0-80.7]	64.5 [34.5-93.2]	47.0 [26.0-75.0]	**0.011**
LDH (U/L)	603.0 [476.0-770.0]	565.0 [466.0-714.0]	627.0 [513.7-793.7]	607.0 [467.0-806.0]	0.674
hs-cTnI (pg/mL)	7.8 [3.0-28.3]	10.0 [4.1-50.6]	7.1 [2.5-28.4]	6.7 [1.6-23.3]	0.337
AST (U/L)	53.5 [37.0-67.0]	59.0 [38.0-78.2]	52.5 [42.0-78.2]	54.0 [39.2-71.0]	0.508
ALT (U/L)	35.5 [25.7-48.2]	39.5 [30.7-50.5]	43.5 [28.0-59.0]	38.5 [29.2-59.0]	**0.023**
ALP (U/L)	170.5 [129.5-	210.0 [156.0-	176.5 [142.7-	178.0 [139.5-	**0.037**

217.2]	274.2]	228.5]	240.0]

Abbreviations: ALP: alkaline phosphatase; ALT: alanine aminotransferase; ARB: angiotensin II receptor blockers; AST: aspartate aminotransferase; BUN: blood urea nitrogen; CRP: C-reactive protein; DM: diabetes mellitus; ESR: erythrocyte sedimentation rate; hs-cTnI: high-sensitivity cardiac troponin I; LDH: lactate dehydrogenase; RBC: red blood cells; SII: systemic immune-inflammation index; WBC: white blood cells.

* Data are presented as mean±standard deviation, number (%), or median [interquartile range].

† Statistically significant P-values are bolded.

‡ Significant post-hoc P-value.

Table S4. Comparison of clinical outcomes of hypertensive patients with COVID-19 based on the history of ARB usage.

**Clinical outcomes***	**Continued (N=186)**	**Discontinued (N=55)**	**Newly started (N=93)**	**Never used (N=227)**	**P†**
Hospital length of stay (day)	6.5 [4.0-10.0]	4.0 [2.0-8.0]	7.0 [4.0-12.0]	4.0 [2.0-7.0]	**<0.001**
In-hospital mortality	38 (20.4%)	25 (45.5%)‡	16 (17.2%)	56 (24.7%)	**0.001**
7-month all-cause mortality	43 (23.1%)‡	28 (50.9%)‡	25 (26.9%)	80 (35.2%)	**<0.001**
ARDS	64 (34.4%)	25 (45.5%)	35 (37.6%)	61 (26.9%)	**0.032**
Invasive ventilation	31 (16.7%)	15 (27.3%)	15 (16.1%)	36 (15.9%)	0.231
ACI	59 (31.7%)	19 (34.5%)	32 (34.4%)	57 (25.1%)	0.235
AKI	43 (23.1%)	14 (25.5%)	17 (18.3%)	28 (12.3%)‡	**0.017**
ALI	20 (10.8%)	8 (14.5%)	12 (12.9%)	25 (11.0%)	0.843
Multiorgan damage	52 (28.0%)	20 (36.4%)	31 (33.3%)	47 (20.7%)	**0.029**

Abbreviations: ACI: acute cardiac injury; AKI: acute kidney injury; ALI: acute liver injury; ARB: angiotensin II receptor blockers; ARDS: acute respiratory distress syndrome.

* Data are presented as number (%) or median [interquartile range].

† Statistically significant P-values are bolded.

‡ Chi-square post-hoc P-value<0.00625.
